# Success Rates of Monitoring for Healthcare Professionals with a Substance Use Disorder: A Meta-Analysis

**DOI:** 10.3390/jcm10020264

**Published:** 2021-01-13

**Authors:** Pauline M. Geuijen, Sophie J. M. van den Broek, Boukje A. G. Dijkstra, Joanneke M. Kuppens, Hein A. de Haan, Cornelis A. J. de Jong, Aart H. Schene, Femke Atsma, Arnt F. A. Schellekens

**Affiliations:** 1 Centre for Neuroscience, Department of Psychiatry, Donders Institute for Brain, Cognition and Behaviour, Radboud University Medical Center, 6500 HB Nijmegen, The Netherlands; Sophie.vandenBroek@radboudumc.nl (S.J.M.v.d.B.); Aart.Schene@radboudumc.nl (A.H.S.); Arnt.Schellekens@radboudumc.nl (A.F.A.S.); 2 Nijmegen Institute for Scientist-Practitioners in Addiction (NISPA), 6525 HR Nijmegen, The Netherlands; boukje.dijkstra@outlook.com (B.A.G.D.); H.deHaan@Tactus.nl (H.A.d.H.); nispa.dejong@gmail.com (C.A.J.d.J.); 3 Novadic-Kentron Addiction Care Network, 5261 LX Vught, The Netherlands; 4 Behavioral Science Institute, Radboud University, 6500 HE Nijmegen, The Netherlands; 5 Royal Dutch Medical Association (RDMA), 3528 BL Utrecht, The Netherlands; joanneke.kuppens@gmail.com; 6 Tactus Addiction Treatment, 7400 AD Deventer, The Netherlands; 7 Scientific Center for Quality of Healthcare (IQ Healthcare), Radboud Institute for Health, Radboud University Medical Center, Sciences, 6500 HB Nijmegen, The Netherlands; Femke.Atsma@radboudumc.nl

**Keywords:** abstinence, healthcare professional, meta-analysis, monitoring, substance use disorder, work retention

## Abstract

In the past decades, monitoring programs have been developed for healthcare professionals with substance use disorders. We aimed to explore estimates of abstinence and work retention rates after participation in such monitoring programs. A literature search was performed using PubMed, Embase, PsycINFO, and CINAHL. Twenty-nine observational studies reporting on success rates (abstinence and work retention) of monitoring for healthcare professionals with a substance use disorder were included in the meta-analysis. Quality-effects models calculated pooled success rates and corresponding 95%-Confidence Intervals (CI), with subgroup analyses on monitoring elements and patient characteristics. Pooled success rates were 72% for abstinence (95%-CI = 63–80%) and 77% for work retention (95%-CI = 61–90%). Heterogeneity across studies was partly explained by the starting moment of monitoring, showing higher abstinence rates for studies that started monitoring after treatment completion (79%; 95%-CI = 72–85%) compared to studies that started monitoring with treatment initiation (61%; 95%-CI = 50–72%). About three-quarters of healthcare professionals with substance use disorders participating in monitoring programs are abstinent during follow-up and working at the end of the follow-up period. Due to selection and publication bias, no firm conclusions can be drawn about the effectiveness of monitoring for healthcare professionals with SUD.

## 1. Introduction

Substance Use Disorders (SUD) are a major health burden, also among healthcare providers, not only affecting their own health, but also their professional image and potentially patient safety [1,2]. Although the prevalence of SUD in healthcare professionals is estimated to be similar to that in the general population (about 10%) [1,3] they more often abuse alcohol and addictive medication, like sedatives and opioids, compared to other SUD patients [4,5].

In the 1970s, the first so-called Physician Health Programs (PHPs) were initiated in the United States. PHPs aim to facilitate early identification and adequate treatment of psychiatric disorders, including SUD, among physicians [6]. Subsequently, health programs were established for other healthcare disciplines and in many more, mainly Western, countries across the globe [7,8]. The content and scope of these health programs vary widely. In the United States (US), professionals are commonly referred to inpatient and/or outpatient treatment in regular care and participate in monitoring provided by the health program [9]. In Europe, some programs mainly provide advice, others provide treatment themselves, and some offer monitoring [7]. A key difference between US health programs and some European programs (e.g., in Norway, Spain, and the United Kingdom (UK)), is that European programs encourage voluntary help seeking by offering free services and have high rates of self-referrals (45–75%) [7]. Additionally, the UK program also guarantees confidentiality by not having any formal links with regulating authorities [10].

Monitoring offers the opportunity to follow the rehabilitation of healthcare professionals with SUD by using biological testing as an objective measure for substance use or abstinence [11]. Monitoring can be started simultaneously with treatment, as well as after successful treatment completion. In addition to biological monitoring of substance use, health programs might also monitor a participants’ fitness to practice at work (by an employer or colleague) or require participation in self-help groups. Health programs usually report outcomes of rehabilitation in terms of abstinence or relapse, return to clinical practice, and/or program completion. A systematic review on rehabilitation outcomes for healthcare professionals found a variety of success rates: abstinence rates of 56% to 94% and work retention rates at the end of follow-up of 74% to 90% [12]. Previous research suggests that this variation in success rates might be influenced by both monitoring elements and participant characteristics [12,13]. Unfortunately, success rates in the systematic review were only presented as ranges per outcome and no thorough examination of the actual data was performed.

So far, there is no meta-analysis performed about success rates of monitoring for healthcare professionals with SUD. Therefore, the current meta-analysis aims to explore success rates of monitoring, using biological testing, for healthcare professionals with SUD, in terms of abstinence and work retention. Furthermore, we explored whether specific monitoring elements and/or participant characteristics explained heterogeneity in success rates across studies.

## 2. Materials and Methods

### 2.1. Search Strategy and Selection Criteria

For this meta-analysis, a review protocol was written, but not published or preregistered before the review was conducted. This protocol adopted a broad search strategy in order to maximize identification of potentially relevant papers. The search strategy, including the definition of outcome measures, was based on a set of a priori identified publications on outcomes of PHPs. The search strategy was developed by a multidisciplinary team with expertise in bibliography (medical librarian), epidemiology (P.M.G., S.J.M.v.d.B., F.A.), and addiction studies (B.A.G.D., A.F.A.S.). The search was performed on 8 December 2020 using the following databases: PubMed, Embase, PsycINFO, and CINAHL.

To be eligible, studies were required to (1) aim at adult healthcare professionals with a SUD diagnosis, (2) clearly describe their (biological) monitoring, and (3) use well-defined outcome measures in terms of abstinence (no relapse during the follow-up period) and/or work retention (working at the end of the follow-up period). Studies were excluded if (1) they concerned tobacco use disorder only, (2) no biological testing was applied, (3) the study solely reported on outcomes of care as usual, or (4) when outcomes were assessed by surveying third parties (i.e., a survey distributed among anesthesia program directors). Studies were limited to English-language research articles published in peer-reviewed journals. Details of the search strategy can be found in Table 1.

### 2.2. Study Selection, Data Extraction, and Quality Assessment

**Figure 1:**
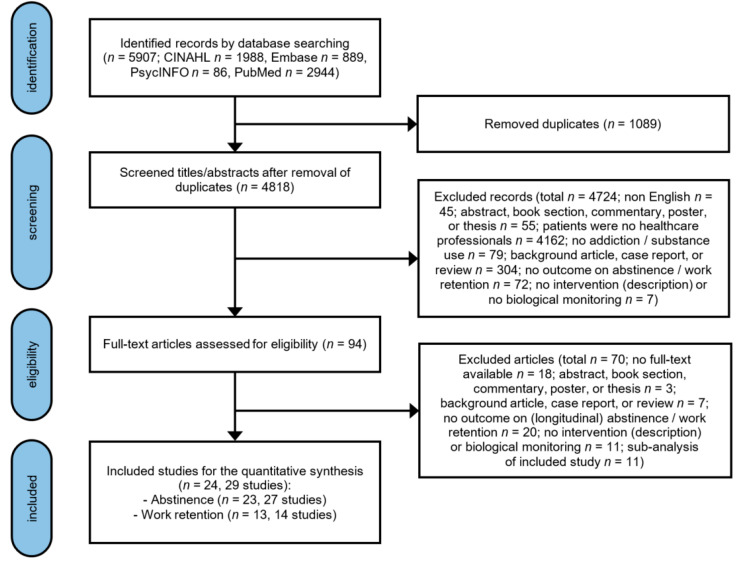
PRISMA flowchart of the selection of studies.

A flow chart of the study selection procedure is provided in Figure 1. First, duplicates were removed, using Rayyan software (Qatar Computing Research Institute, Doha, Qatar, 2017) for citation screening [14]. Next, three authors (P.M.G., S.J.M.v.d.B., and B.A.G.D.) screened 5907 unique titles and abstracts on the selection criteria mentioned above. Discrepancies in the identified eligible records were discussed until consensus was reached. When in doubt, records moved on to the next phase of assessing the eligibility, based on the full-text articles. Full-text assessment of 94 remaining records was performed by two authors (P.M.G. and S.J.M.v.d.B.). Discrepancies were discussed until consensus was reached. This resulted in 29 studies eligible for the meta-analysis, published in 24 articles. Next, data-extraction was performed by one researcher (P.M.G.). The data of each study was documented in Microsoft Excel 2016, which was subsequently checked by a second researcher (S.J.M.v.d.B).

Extracted information included study characteristics: name of first author, year of publication, country (state) of first author, design of the study, time frame of the study, number of included subjects, percentage of males, type of healthcare professional, type of substance use, and source of referral. In addition, characteristics of monitoring were summarized: name of the health program, recommended type of treatment, starting moment of monitoring, type of biological testing, monitoring at work, and additional agreements. Finally, the outcomes of monitoring programs were extracted: percentage of abstinence and work retention specified with the (exact or range of) duration of follow-up. Since the information was not always presented in the same manner, we categorized monitoring elements and participant characteristics in order to perform subgroup analyses: program elements (biological, at work, and additional agreements; biological and additional agreements; biological and at work; biological), starting moment of monitoring (before treatment; after treatment; unknown), duration of follow-up (less than 2 years; 2 to 5 years; more than 5 years; other duration), gender (more than 50% males; other or unknown), type of healthcare professional (more than 50% physicians; other or mixed), and type of substance use (more than 50% alcohol; more than 50% opioids; mixed or unknown).

All included studies were assessed on their quality in order to account for study quality in the meta-analysis. The initial assessment was performed by one researcher (P.M.G.), and subsequently checked by a second researcher (S.J.M.v.d.B.). The Health States Quality Index [15] was used to assess study quality. Assessment parameters include a clear definition of the target population and observation period (yes or no), use of diagnostic criteria (diagnostic system or symptom based/not specified), method of case selection (attempting all cases, convenience sampling, or not specified), type of outcome assessment (administered interview, register/case record, or not specified), size of the study area (broad, small, or not specified), and type of prevalence measure (exact follow-up duration, average follow-up duration, or range of follow-up duration). The quality index of each study is calculated as the total quality score of that study divided by the maximum total quality score, see Table A1. The instrument was slightly adjusted for a good fit to our meta-analysis. The higher the score, the higher the study quality. We report our study in accordance with the Preferred Reporting Items for Systematic reviews and Meta-Analyses (PRISMA) and the proposal for reporting Meta-analyses of Observational Studies in Epidemiology (MOOSE) were applicable, see Table S1 [16,17].

### 2.3. Data-Analysis

Statistical analyses were performed using MetaXL (EpiGear International Pty Ltd., Sunrise Beach, Australia, version 5.3) within Microsoft Excel 2016 [15,18]. For every study, the total number of participants, the number of participants with a successful outcome (abstinence or work retention), and the quality index were entered in MetaXL. Quality-effects models were used in order to address heterogeneity caused by differences in study quality. The quality-effects model is a modified version of the fixed-effects inverse variance method and gives greater weight to the studies that were judged as being of high quality [19]. The models were applied to analyze the data and calculate pooled abstinence and work retention rates, and accompanying 95%-Confidence Intervals (CI).

The heterogeneity assumption was assessed by Cochrane’s Q-test (which verifies the presence of heterogeneity) and I2 statistic (which shows the amount of heterogeneity between studies). A significant Q-test (*p* < 0.10) and an I2 > 50% indicated the presence of substantial heterogeneity. Subgroup analyses were explored by stratifying the data on monitoring elements (start of monitoring, type of monitoring, and duration of follow-up) and participant characteristics (gender, type of healthcare professional, and type of substance use).

Publication bias was assessed using the Doi plot and Luis Furuya-Kanamori asymmetry (LFK) index. In the case of a symmetric shape, no publication bias is indicated. In case of an asymmetric shape, publication bias is indicated. An LFK index within −1 and +1 indicates no publication bias, an LFK of −1 to −2 or +1 to +2 minor asymmetry, and an LFK of <−2 or >+2 major asymmetry [15].

## 3. Results

### 3.1. Description of the Included Studies

The study and monitoring characteristics of the 29 included studies (out of 24 articles) are summarized in Table 2 and Table 3. Studies were published between 1982 and 2020 and mainly conducted in the United States, one in Canada, one in Australia, and one in Spain. The design of most studies was observational (either retrospective or prospective data collection). One study had a descriptive design (survey among healthcare professionals engaged in a monitoring program), and one performed an experiment (single-arm multisite, open label study of injectable naltrexone in healthcare professionals with opioid dependence). None of the included studies used randomized controlled trial or quasi-experimental designs.

About half of the studies mainly included male subjects (14 studies) and physicians only (20 studies). The most commonly reported substances of abuse were opioids (22 studies) and alcohol (16 studies). Studies that indicated the source of referral to monitoring reported licensing boards (8 studies), employers (5 studies), colleagues (7 studies), family members (6 studies), treatment providers (2 studies), and self-referrals (9 studies). All health programs offered biological monitoring, whether or not in combination with monitoring at work (10 studies), and/or other monitoring arrangements (28 studies). Monitoring started either simultaneously with treatment initiation (8 studies) or after treatment completion (17 studies). Four studies did not indicate when monitoring started. Sample size varied widely between 11 to 904 healthcare professionals, with data available for a total of 3027 healthcare professionals for abstinence, and 1728 for work retention. Follow-up range also varied widely between 0 to 8 years; including 5 studies on abstinence and 2 studies on work retention with a follow-up of 5 years or more. The quality index of the included studies ranged from 0.2 to 0.9, see Table A1.

### 3.2. Abstinence

**Figure 2:**
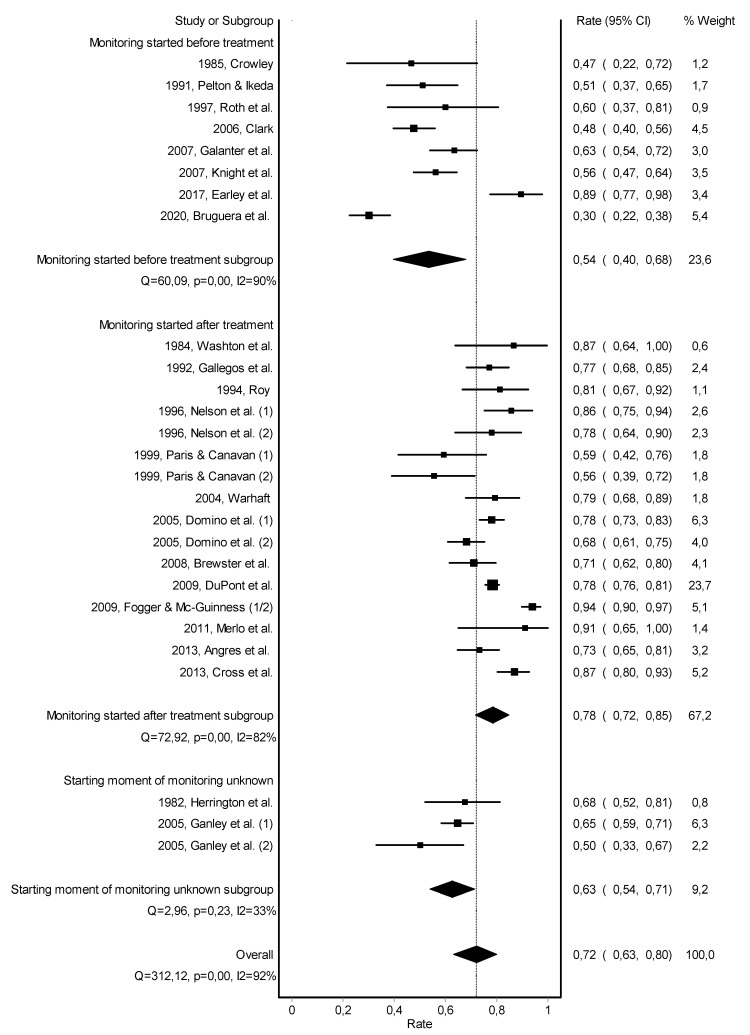
Forest plot of the pooled abstinence rate—subgroup analysis based on starting moment of monitoring.

Abstinence rates in the individual studies ranged from 30 to 94% with a substantial heterogeneity across studies (Q = 312.1; *p* < 0.001; I2 = 92%). The overall pooled abstinence rate across studies was 72% (95%-CI = 63−80%), with a follow-up duration up to 8 years, see Figure 2. When stratified by starting moment of monitoring, the subgroup analysis slightly reduced heterogeneity across studies and showed a higher abstinence rate among studies that started monitoring after treatment completion (79%; 95%-CI = 72–85%; Q = 74.0; *p* < 0.001; I2 = 80%), compared to studies that started monitoring at treatment initiation (53%; 95%-CI = 40–67%; Q = 60.3; *p* < 0.001; I2 = 88%).

**Figure A1:**
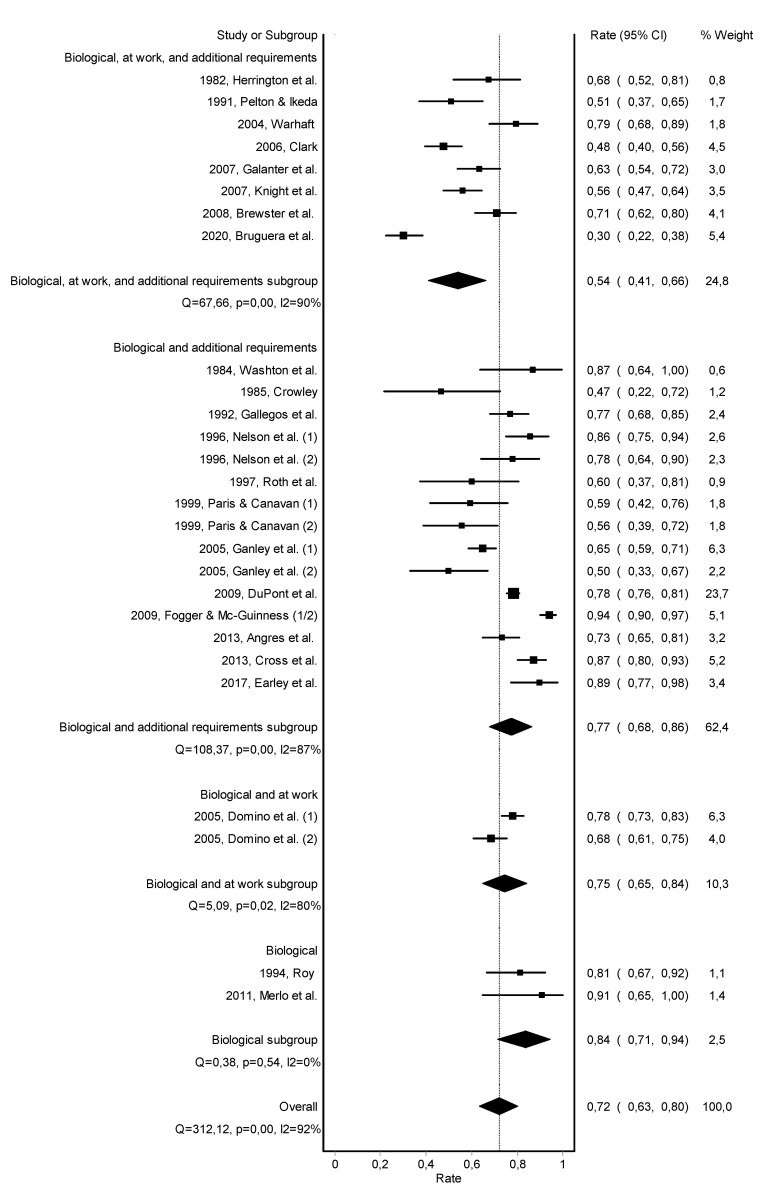
Forest plot of the pooled abstinence rate—subgroup analysis based on type of monitoring.

**Figure A2:**
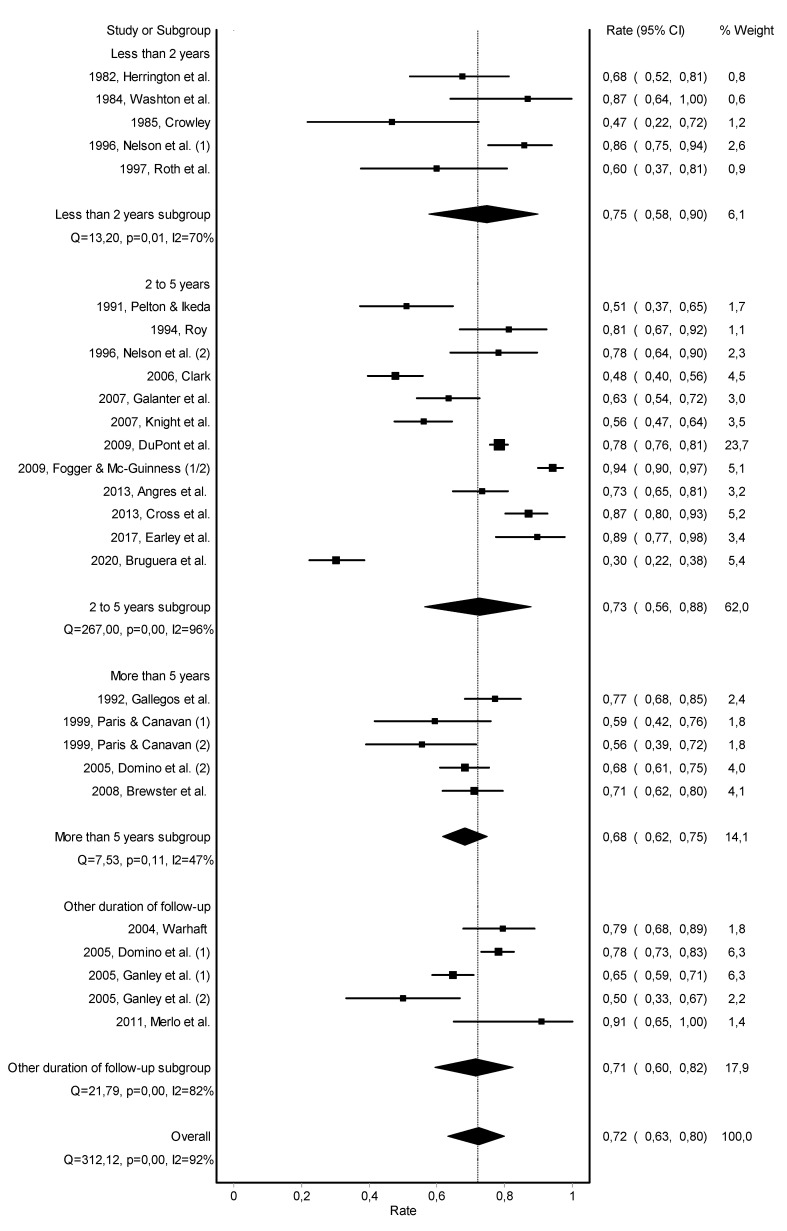
Forest plot of the pooled abstinence rate—subgroup analysis based on duration of follow-up.

**Figure A3:**
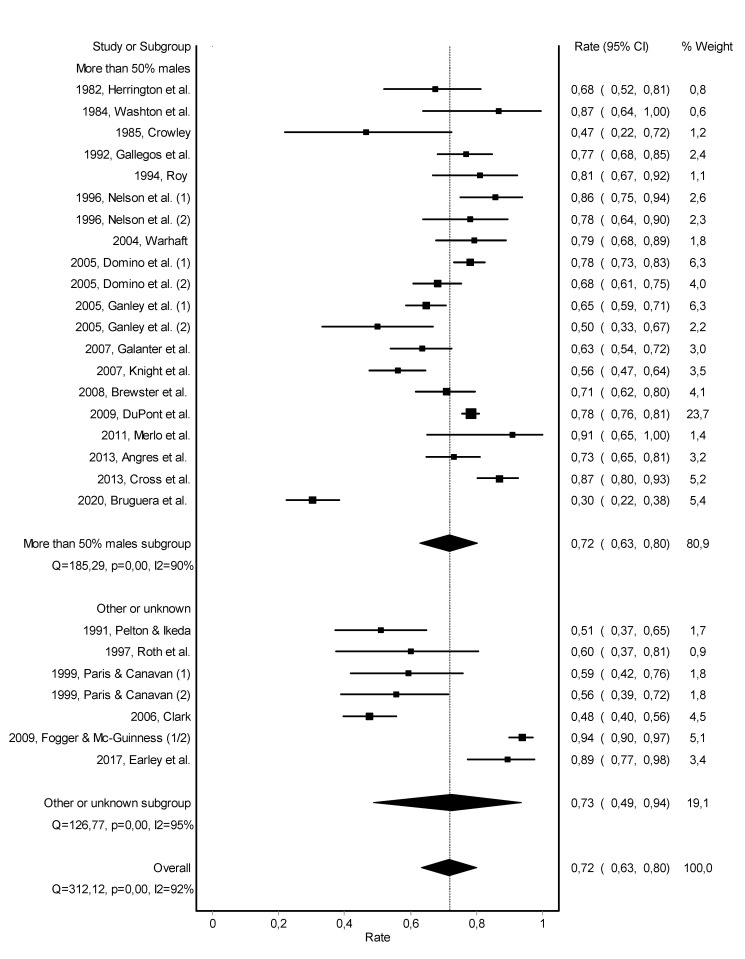
Forest plot of the pooled abstinence rate—subgroup analysis based on gender.

**Figure A4:**
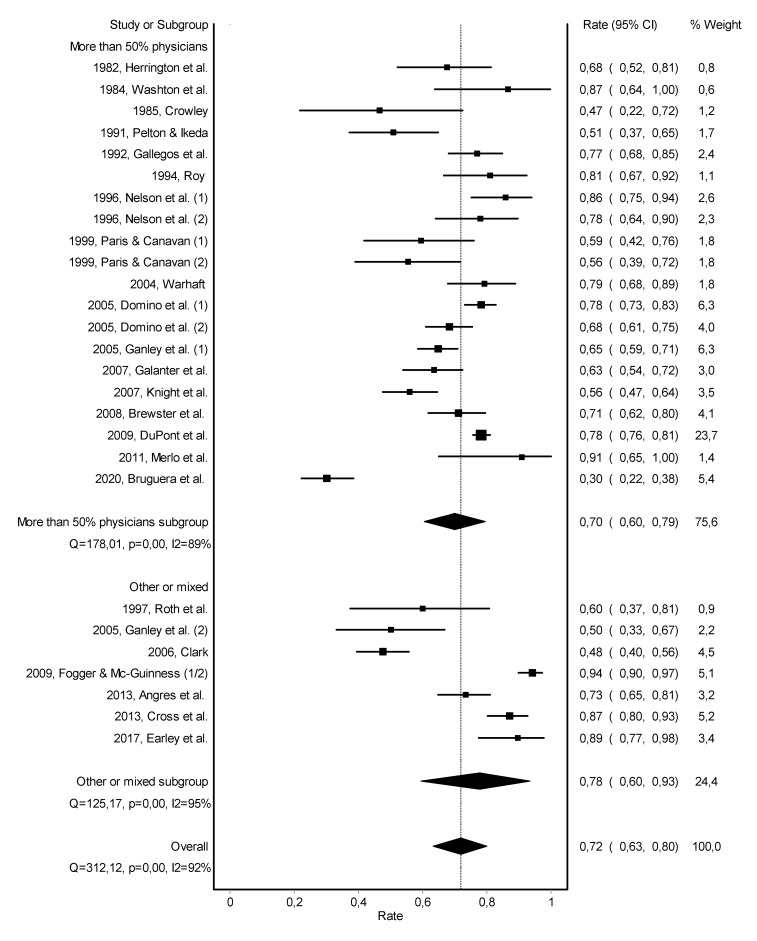
Forest plot of the pooled abstinence rate—subgroup analysis based on type of healthcare professional.

**Figure A5:**
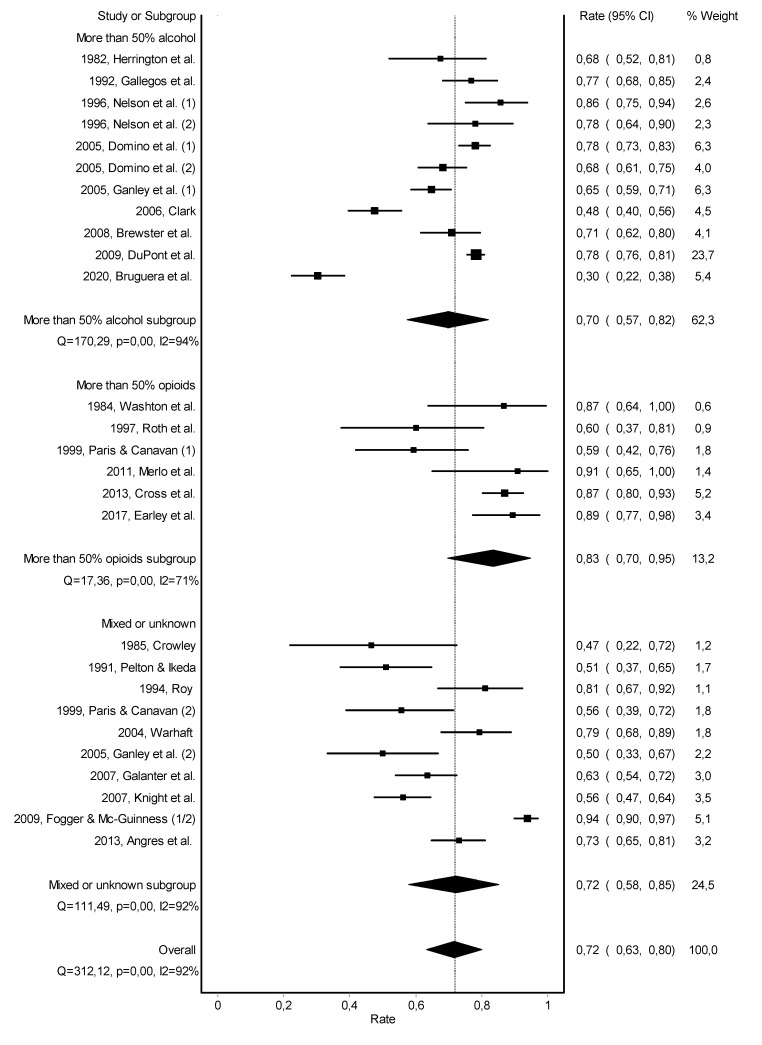
Forest plot of the pooled abstinence rate—subgroup analysis based on type of substance use.

**Figure A6:**
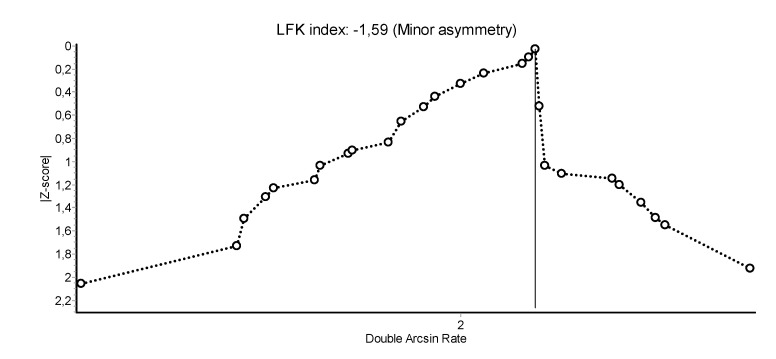
Doi plot analysis and Luis Furuya-Kanamori asymmetry (LFK) index of publication bias for the pooled abstinence rate.

Subgroup analyses on the type of monitoring did slightly reduce heterogeneity across studies (Figure A1). Heterogeneity across studies was not significantly reduced by duration of follow-up, gender, type of healthcare professional, and type of substance use (Figure A2, Figure A3, Figure A4 and Figure A5). Risk of bias across studies was visualized in a Doi plot, indicating an asymmetric shape for the pooled abstinence rate (Figure A6). The LFK index was −1.59, also indicating minor publication bias.

### 3.3. Work Retention

**Figure 3:**
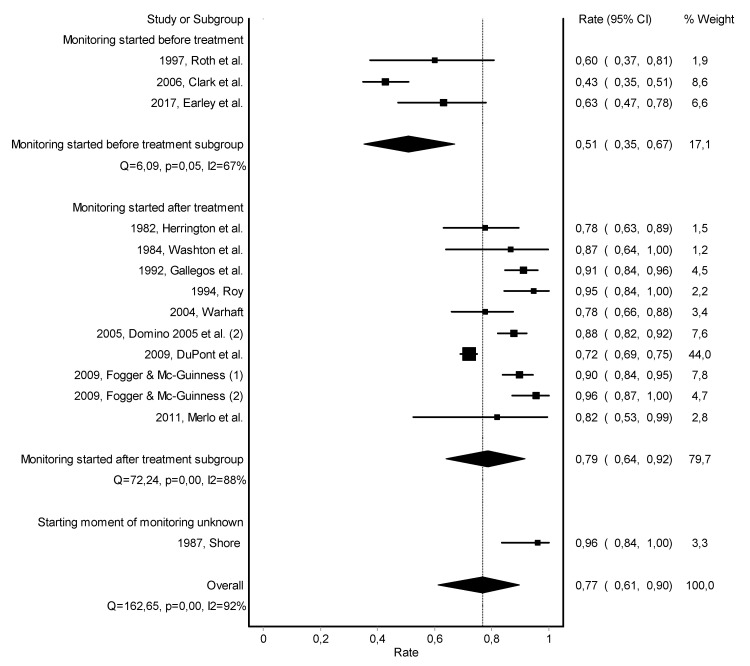
Forest plot of the pooled work retention rate—subgroup analysis based on starting moment of monitoring.

Work retention rates of the individual studies ranged from 43 to 96% with a substantial heterogeneity across studies (Q = 162.7; *p* < 0.001; I2 = 92%). The overall pooled work retention rate was 77% (95%-CI = 61–90%), with a follow-up duration up to 8 years (Figure 3).

**Figure A7:**
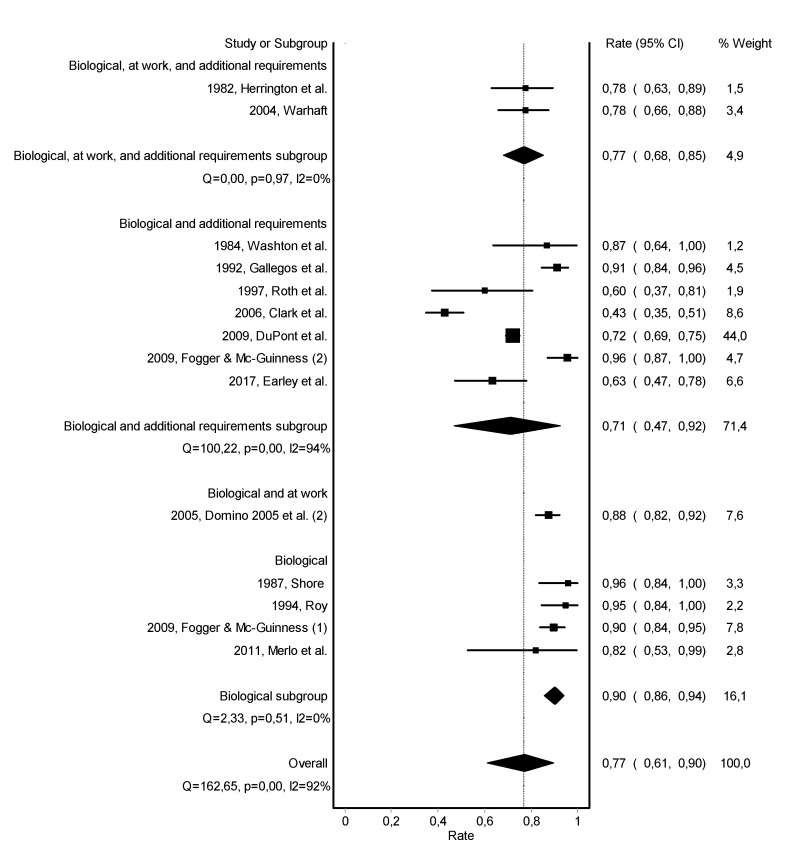
Forest plot of the pooled work retention rate—subgroup analysis based on type of monitoring.

**Figure A8:**
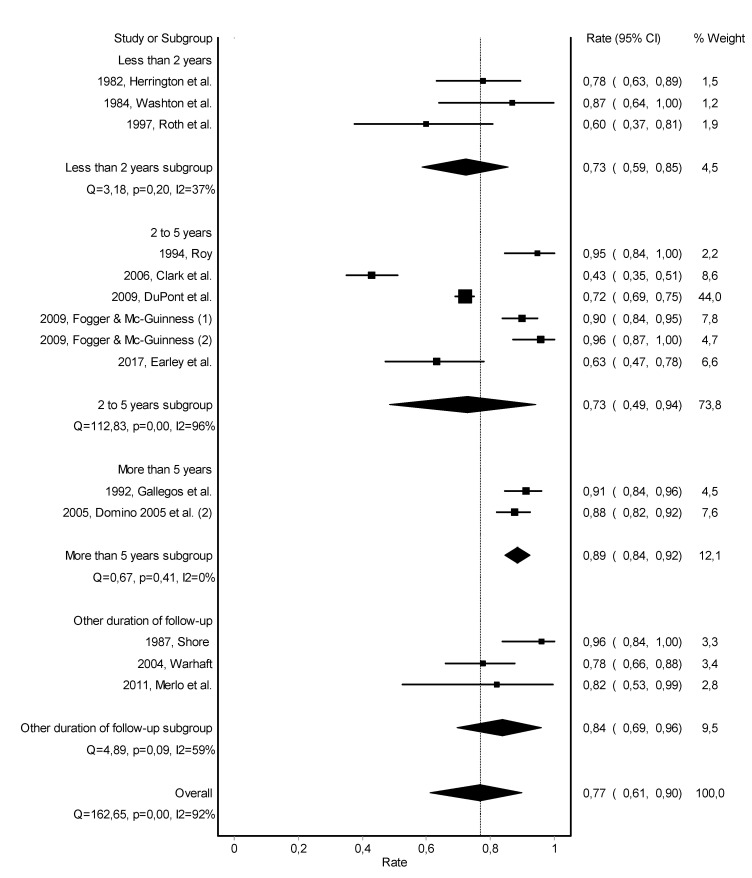
Forest plot of the pooled work retention rate—subgroup analysis based on duration of follow-up.

**Figure A9:**
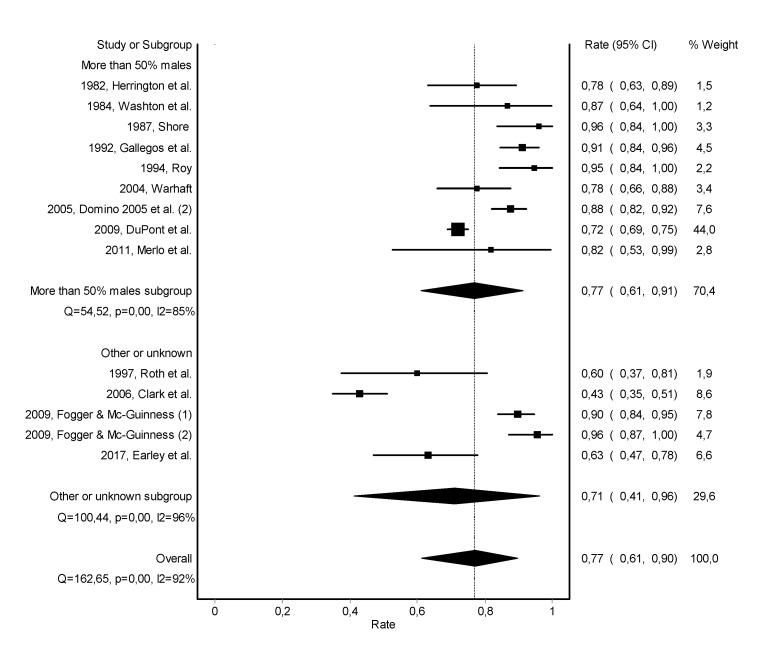
Forest plot of the pooled work retention rate—subgroup analysis based on gender.

**Figure A10:**
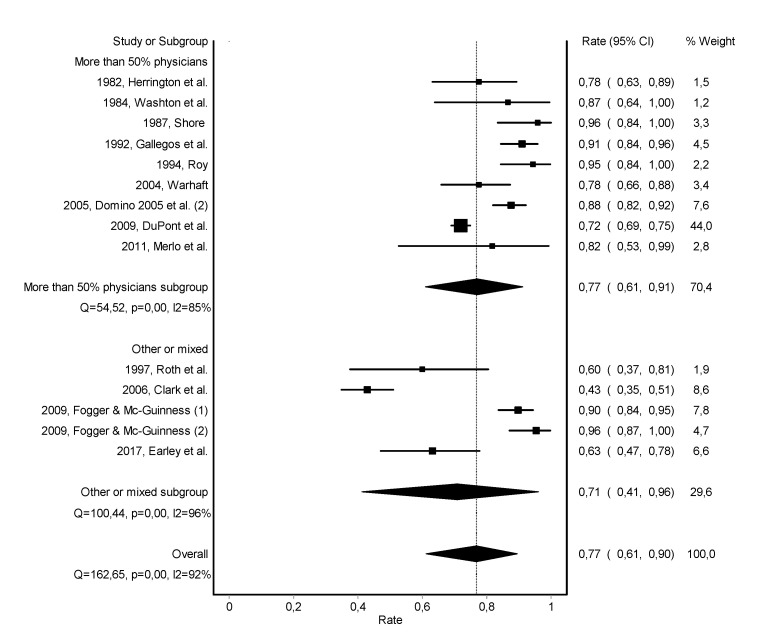
Forest plot of the pooled work retention rate—subgroup analysis based on type of healthcare professional.

**Figure A11:**
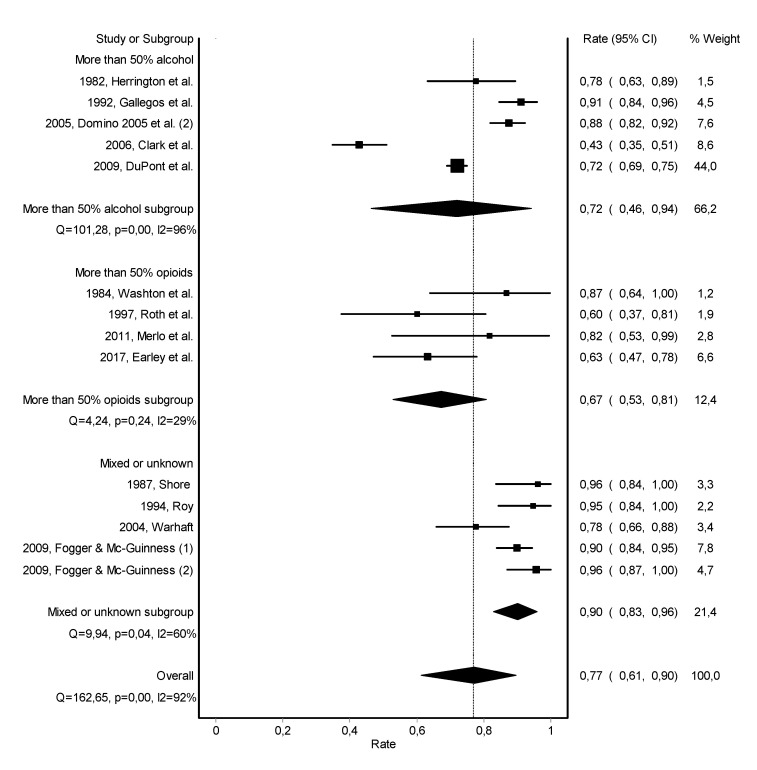
Forest plot of the pooled work retention rate—subgroup analysis based on type of substance use.

**Figure A12:**
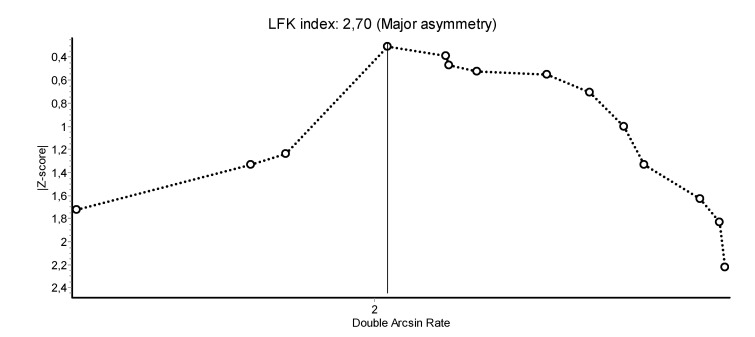
Doi plot analysis and Luis Furuya-Kanamori asymmetry (LFK) index of publication bias for the pooled work retention rate.

Subgroup analyses on type of monitoring and type of substance use did slightly reduce heterogeneity across studies (Figure A7 and Figure A11). Subgroup analyses on starting moment of monitoring, duration of follow-up, gender, and type of healthcare professional did not significantly reduce heterogeneity across studies (Figure 3, Figure A8, Figure A9 and Figure A10). Risk of bias across studies was visualized in a Doi plot, indicating an asymmetric shape for the pooled work retention rate (Figure A12). The LFK index was −2.70, also indicating major publication bias.

## 4. Discussion

This study aimed to identify the success rate of monitoring for healthcare professionals with SUD, as indexed by abstinence and work retention. Furthermore, possible explaining variables for heterogeneity were explored. On average, three quarters of the healthcare professionals who engaged in a monitoring program remained abstinent and were working at follow-up. Follow-up duration varied widely between 0 to 8 years. We identified significant heterogeneity across studies, as well as indication for publication bias. Heterogeneity within abstinence rates was partly explained by the starting moment of monitoring. Monitoring that started after successful initial treatment had better outcomes compared to those that started monitoring simultaneously with treatment. Duration of follow-up, gender, and type of healthcare professional did not significantly decrease the heterogeneity in success rates.

Unfortunately, none of the included studies used a randomized control trial or quasi-experimental design, and due to the naturalistic design of the studies included in this meta-analysis we cannot draw firm conclusions on the effectiveness of monitoring programs for healthcare professionals with SUD. If the actual effectiveness of monitoring turns out to be comparable to the success rates we found, this would be promising. In general, SUD patients show relapse rates over 50% within the first year after treatment initiation, and they remain at increased risk for relapse throughout the early years of recovery [42,43,44]. Professionals in monitoring were thus about 1.5 times more successful in maintaining abstinence when compared to regular addiction care patients without monitoring. Biological monitoring has also been applied in general SUD patients, showing a one-year abstinence rate of 46% [45,46]. This is far less successful as observed here among healthcare professionals. This may be partly attributed to the starting moment of monitoring (during treatment), but might also be the result of a difference in effectiveness of the intervention. Furthermore, work retention is a major incentive for healthcare professionals, which might apply to a lesser extent in general SUD patients. Indeed, studies on Contingency Management (CM) and Community Reinforcement Approach (CRA) indicate that positive reinforcement increases abstinence rates [47].

We only included studies that applied biological monitoring of substance use. Biological testing is the most reliable and objective measure for abstinence [11]. The studies included in this meta-analysis mostly reported urine toxicology as method of biological testing. Yet, abstinence rates might be inflated due to false-negative urine toxicology [48]. On the other hand, biological testing might be more effective in promoting abstinence than self-report. Indeed, studies on monitoring without biological testing among healthcare professionals showed somewhat less positive results (i.e., abstinence rates ranging from 13% to 76% and work retention rates ranging from 36% to 89%) [49,50,51,52,53,54,55,56,57,58]. This might indicate that monitoring programs should preferably include biological monitoring of substance use.

Heterogeneity in abstinence rates across studies was partially explained by the starting moment of monitoring. This suggests a potential source of selection bias, depending on the timing of monitoring. Participants who start monitoring after successful treatment completion might be strongly motivated to achieve abstinence and have high chances to maintain their good treatment outcome. Moreover, the group who starts monitoring simultaneously with treatment initiation also includes participants who will drop out of treatment, or relapse during treatment. This will lead to lower success rates of monitoring. Indeed, many continuing care studies limited their participants to those who had successfully completed the initial treatment phase, thus introducing selection bias [59]. Other variables included in the subgroup analyses (duration of follow-up, gender, and type of healthcare professional) did not explain a substantial part of the heterogeneity across studies. Unfortunately, the data reported in the included studies did not enable us to perform subgroup analyses on type of initial treatment (inpatient, outpatient, pharmacological intervention, etc.) and on the mandatory status of monitoring.

Several other sources of bias might affect our findings. First, it has been suggested that many physicians who are forced to participate in a PHP might not actually have a SUD [60]. Not all PHPs use diagnostic criteria to assess their participants. Indeed, more than two-thirds of the studies included in our meta-analysis did not specify the diagnostic process of SUD assessment. Secondly, some of the studies we included did not take into account participants who were lost to follow-up in calculating the overall success rate of monitoring. It is unclear how this may have influenced the outcomes. Participants may have become lost to follow-up either because they are doing well and feel they no longer need monitoring or, on the other end of the spectrum, because they have relapsed and cannot be located or do not want to reveal their condition [59]. Thirdly, the duration of follow-up varied widely within and between the included studies and durations were either presented as range, average, or exact follow-up between 0 to 8 years. A follow-up of 0 years meant that some participants recently started monitoring, whereas other participants in the same study were followed-up for 3 or 5 years. Fourthly, three very small studies either showed high [21,37] or low [22] success rates, thereby possibly skewing the results. Though some success rates changed slightly, the sensitivity analyses showed that the main findings still hold, indicating the robustness of findings. Lastly, our meta-analysis showed asymmetry for both abstinence and work retention, suggesting publication bias. Taken together, this raises concerns of potential overestimation of the effectiveness of monitoring in the current literature [60]. In order to reduce reporting and publication bias, we strongly encourage health programs to systematically assess effectiveness and publish about the outcomes of their monitoring.

The current study results should be interpreted in the light of several limitations. First, we identified a considerable amount of heterogeneity between studies, but were able to explain only a small fraction by the starting moment of monitoring. Other potential sources of heterogeneity like the severity of the SUD, the presence of comorbidity, a (family) history of SUD, the type of initial treatment (inpatient, outpatient, pharmacological and/or psychological intervention), and the status of monitoring (mandatory or voluntary) could not be analyzed since this information was generally not available across studies [31,33,61]. Secondly, we included only English-language research articles published in peer-reviewed journals. This might have increased bias in our study results, because we did not include foreign language studies, unpublished studies, partially published studies, and studies published in “grey” literature sources [62]. Thirdly, the definition of the abstinence outcome measure (no relapse during follow-up) was quite strict, so some abstinence rates included in the meta-analysis were lower than reported in the conclusions of the individual studies. Furthermore, the overall quality of the included studies was moderate, with 60% of the studies scoring 0.5 or lower on the Quality Index. Thus, future studies with more rigorous designs are highly needed, in order to support effectiveness of monitoring for healthcare professionals with SUD. Finally, we focused only on healthcare professionals with SUD. Therefore, we cannot say anything about behavioral addictions or other psychiatric problems among healthcare professionals. Yet, some studies investigated the success of monitoring for other psychiatric problems among healthcare professionals, showing high recovery rates ranging from 88 to 94% and work retention rates ranging from 77 to 90% [12]. The current positive findings may thus indicate good prognosis of mental health issues in general among healthcare professionals.

## 5. Conclusions

Three quarters of the healthcare professionals who engaged in monitoring for SUD remained abstinent and were working at follow-up. There was significant heterogeneity across studies, as well as an indication for major publication bias. The heterogeneity in success rates of monitoring was slightly explained by the starting moment of monitoring, with studies starting monitoring after treatment completion showing higher success rates than studies starting monitoring at treatment initiation. Given the heterogeneity across studies and indication for publication bias, no firm conclusions can be drawn about the effectiveness of monitoring for healthcare professionals with SUD. Future studies should apply controlled comparisons, using more rigorous measurements and substantially long follow-up rates.

## Figures and Tables

**Table 1:** Search strategy.

Population	((“Health personnel”[MeSH] OR “Medical staff”[MeSH] OR Dentist*[tiab] OR Doctor*[tiab] OR General practitioner*[tiab] OR Health personnel[tiab] OR Healthcare personnel[tiab] OR Healthcare provider*[tiab] OR Healthcare professional*[tiab] OR Medical staff[tiab] OR Nurse*[tiab] OR Nursing staff[tiab] OR Pharmacist*[tiab] OR Physician*[tiab] OR Physician assistant*[tiab]) AND (“Alcohol-related disorders”[MeSH] OR “Alcoholism”[MeSH] OR “Opioid-related disorders”[MeSH] OR “Substance-related disorders”[MeSH] OR Alcohol abuse*[tiab] OR Alcohol addict*[tiab] OR Alcohol dependen*[tiab] OR Alcohol impair*[tiab] OR Alcohol misuse[tiab] OR Alcohol use disorder*[tiab] OR Alcohol-related disorders[tiab] OR Alcoholism[tiab] OR Drug abuse*[tiab] OR Drug addict*[tiab] OR Drug dependen*[tiab] OR Drug impair*[tiab] OR Drug misuse[tiab] OR Drug use disorder*[tiab] OR Opiate abuse*[tiab] OR Opioid abuse*[tiab] OR Opiate addict*[tiab] OR Opioid addict*[tiab] OR Opiate dependen*[tiab] OR Opioid dependen*[tiab] OR Opiate impair*[tiab] OR Opioid impair*[tiab] OR Opiate misuse[tiab] OR Opioid misuse[tiab] OR Opioid-related disorders[tiab] OR Substance abuse*[tiab] OR Substance addict*[tiab] OR Substance dependen*[tiab] OR Substance impair*[tiab] OR Substance misuse[tiab] OR Substance use disorder*[tiab] OR Substance-related disorders[tiab])) OR (“Professional impairment”[MeSH] OR Dentist impair*[tiab] OR Doctor impair*[tiab] OR Nurse impair*[tiab] OR Pharmacist impair*[tiab] OR Physician impair*[tiab] OR Physician assistant impair*[tiab] OR Professional impairment[tiab])
	AND
Intervention	(“Health services”[MeSH] OR “Occupational health”[MeSH] OR “Mental disorders”[MeSH] OR “Referral and consultation”[MeSh] OR Employee assistance program*[tiab] OR Employee health service*[tiab] OR Health agenc*[tiab] OR Health program*[tiab] OR Health service*[tiab] OR Occupational health[tiab] OR Occupational health service*[tiab] OR Mental disorders[tiab] OR Referral and consultation[tiab]) OR (“Biological monitoring”[MeSH] OR “Mental health recovery”[MeSH] OR “Psychiatric rehabilitation”[MeSH] OR Biological monitor*[tiab] OR Mental health rehabilitation[tiab] OR Mental health recovery[tiab] OR Physiologic monitor*[tiab] OR Psychiatric rehabilitation[tiab] OR Psychosocial rehabilitation[tiab] OR Recover*[tiab]) OR (“Self-help groups”[MeSH] OR Self-help group*[tiab] OR Support group*[tiab] OR Alcoholics anonym*[tiab] OR Narcotics anonym*[tiab])
	AND
Outcome	(“Outcome assessment, health care”[MeSH] OR “Program evaluation”[MeSH] OR “Treatment outcome”[MeSH] OR Outcome assessment*[tiab] OR Outcome measure*[tiab] OR Program effect*[tiab] OR Program evaluation[tiab] OR Treatment effect*[tiab] OR Treatment failure*[tiab] OR Treatment outcome*[tiab] OR Recovery rate*[tiab] OR Rehabilitation outcome*[tiab]) OR (“Alcohol abstinence”[MeSH] OR “Recurrence”[MeSH] OR Abstinence[tiab] OR Alcohol abstinence[tiab] OR Drug abstinence[tiab] OR Opioid abstinence[tiab] OR Substance use abstinence[tiab] OR Recurrence[tiab] OR Relapse*[tiab]) OR (“Return to work”[MeSH] OR “Work performance”[MeSH] OR Job perform*[tiab] OR Job retention[tiab] OR Return to work[tiab] OR Work perform*[tiab] OR Work resum*[tiab] OR Work retention[tiab])

* This strategy is related to the PubMed search. Very similar versions were used to search Embase, PsycINFO, and CINAHL, but adapted for the specific search terms used in these databases.

**Table 2:** Study characteristics.

Year, Study Country (State)	Design	Time Frame	Subjects (N)	Males (%)	Type of Healthcare Professional (%)	Type of Substance Use (%)	Source of Referral (%)
1982, Herrington et al. [20] US (Wisconsin)	retrospective review	1979–1982	40	95	general practitioner (28); anesthesiologist (13); psychiatrist (10); internal medicine (8); dentist (8); obstetrics-gynecology (8); surgeon (5); other (20)	alcohol (58); narcotics (38); other (5)	coworker (63); family member (18); legal system (13); self-referral (8)
1984, Washton et al. [21] US (New York; New Jersey)	retrospective review	1979–1981	15	100	physician (100)	opioids (100)	-
1985, Crowley [22] US (Colorado)	prospective descriptive	-	15	100	physician (60); dentist (33); veterinarian (7)	-	licensing board (40); hospital or coworkers (33); family member (7); treatment provider (7); self-referral (13)
1987, Shore [23] US (Oregon)	retrospective review	1977–1985	25	-	physician (100)	-	-
1991, Pelton & Ikeda [24] US (California)	retrospective review	1980–1990	51	-	anesthesiologist (100)	opioids (49)	-
1992, Gallegos et al. [25] US (Georgia)	retrospective review	1982–1992	100	92	family and general practitioner (23); surgeon (22); anesthesiologist (17); psychiatrist (15); internal medicine (12); emergency medicine (4); pediatrician (3); radiologist (1); dermatologist (1); occupational medicine (1); rehabilitation medicine (1)	alcohol (71); cocaine (21); meperidine hydrochloride (19); diazepam (18); marijuana (17); percodan (12); fentanyl citrate (11); codeine sulfate (9); amphetamine (7)	-
1994, Roy [26] US (Louisiana)	retrospective review	>1989	37	89	physician (68); dentist (16); pharmacist (5); veterinarian (3); other (8)	prescription drug (43); alcohol (27); polysubstance (16); cocaine (14)	-
1996, Nelson et al. (1) [27] US (Oregon)	retrospective review	1990–1992	56	91	surgery (59); internal medicine (32); family practitioner (21); emergency medicine (7); anesthesiology (6); pathology (4); pediatrician (4); obstetrics-gynecology (3); psychiatry (2); neurology (2); dermatology (1); radiology (1); unknown (1)	alcohol (75); opioids and cocaine (21); amphetamines and sedatives (4)	self-referral (15); immediate contact (39); third party (46)
1996, Nelson et al. (2) [27] US (Oregon)	retrospective review	1990–1992	41	90		alcohol (87); opioids and cocaine (8); amphetamines and sedatives (5)	self-referral (7); immediate contact (15); third party (73); unknown (5)
1997, Roth et al. [28] US (Connecticut)	retrospective review	-	20	15	nurse (85); anesthesiology nurse (10); pharmacist (5)	opioids (100); alcohol (85); cocaine (40); benzodiazepines (30)	licensing board (90); self-referral (10)
1999, Paris & Canavan (1) [29] US (New Jersey)	retrospective review	1982–1994	32	-	anesthesiologist (59); anesthesiology residents (41)	opioids (78)	-
1999, Paris & Canavan (2) [29] US (New Jersey)	retrospective review	1982–1994	36	-	physician (75); resident (25)	opioids (42)	-
2004, Warhaft [30] Australia	retrospective review	2001–2004	58	86	general practitioner (34); anesthesiologist (10); surgeon (7); pathologist (5); radiologist (5); physician (3); obstetrics-gynecology (2); occupational medicine (2); pediatrician (2); psychiatry (2); other (28)	alcohol (36); pethidine (31); heroin (12); codeine (5); benzodiazepines (5); amphetamines (3); cocaine (3); nitrous oxide (2); ketamine (2)	-
2005, Domino et al. [31] US (Washington)	retrospective cohort	1991–2001	292	84	physician (79); physician assistant (11); veterinarian (5); osteopath (2); dentist/dental surgeon (1); podiatrist/pharmacist (1)	alcohol (56); opioids (32); cocaine (3); benzodiazepines (2); other (7)	-
2005, Ganley et al. (1) [32] US (North Carolina)	retrospective review	1991–2001	233	87	physician (100)	alcohol (50); opioids (25); polysubstance (16); other (8)	licensing board; hospital; coworker; family member; self-referral
2005, Ganley et al. (2) [32] US (North Carolina)	retrospective review	1991–2001	34	74	physician assistant (100)	alcohol (44); opioids (35); polysubstance (6); other (15)	licensing board; hospital; coworker; family member; self-referral
2006, Clark et al. [33] US (Idaho)	retrospective review	1985–2000	147	18	registered nurse (57); licensed practical nurse (38); advanced practice registered nurse (3)	alcohol (72); legal oral opioids (45); inhalants (8); stimulants (23); marijuana (21); legal injected narcotics (31); illegal injected opioids (33); prescription drugs (20)	employer (50); licensing board (14); coworker (6); treatment provider (6); self-referral (14)
2007, Galanter et al. [34] US (New York; Nevada)	retrospective review	2003–2004	104	92	anesthesiologist (21); internal medicine (15); surgeon (14); family practitioner (10); obstetrics-gynecology (9); pediatrician (8); psychiatrist (8); general practitioner (4); emergency medicine (4); radiologist (3); other (5)	alcohol (36); opioids (34); other or mixed (30)	-
2007, Knight et al. [13] US (Massachusetts)	retrospective observations	1993–2003	132	82	internal medicine (31); psychiatrist (12); surgeon (12); anesthesiologist (11); emergency medicine (8); family practitioner (6); obstetrics-gynecology (6); radiologist (4); pediatrician (3); other (6)	-	-
2008, Brewster et al. [35] Canada	prospective descriptive	1995–2007	100	90	general or family practitioner (51); specialist (49)	alcohol (51); opioids (37); other (13)	-
2009, DuPont et al. [9] US (Maryland; Pennsylvania; Indiana; Florida)	retrospective review	1995–2001	904	86	family practitioner (20); internal medicine (13); anesthesiologist (11); emergency medicine (7); psychiatrist (7); other (42)	alcohol (50); opioids (33); stimulants (8); other (9)	licensing board, hospital, malpractice insurance company (55); family member, coworker, employer (45)
2009, Fogger & Mc-Guinness (1) [36] US (Alabama)	cross-sectional survey	-	127	-	registered nurse (77); licensed practical nurse (13); advanced practice registered nurse (8)	opioids (36)	-
2009, Fogger & Mc-Guinness (2) [36] US (Alabama)	cross-sectional survey	-	45	-	registered nurse (78); licensed practical nurse (18); advanced practice registered nurse (4)		-
2011, Merlo et al. [37] US (Florida)	retrospective review	≥ 2005	11	100	anesthesiologist (100)	opioids (100)	-
2013, Angres et al. [38] US (Illinois)	prospective cohort	-	116	68	physician (48); nurse (24); pharmacist (18); dentist (7); optometrist (1); physician assistant (1); other (1)	-	licensing board (100)
2013, Cross et al. [39] US (Illinois)	prospective descriptive	1994–2011	116	78	pharmacist (100)	oral opioids (71); alcohol (22); illegal drugs (9); stimulants (8); injected opioids (3)	-
2017, Earley et al. [40] US (Georgia)	single-arm multisite, open label	2009–2012	38	18	nurse (79); physician (11); pharmacist (3); other (8)	opioids (100)	-
2020, Bruguera et al. [41] Spain	prospective descriptive	2008–2016	126	60	family practitioner (17); psychiatrist (9); anesthesiologist (9); pediatrician (6); orthopedic surgeons (6); internal medicine (3); resident (4); other (47)	alcohol (63); sedatives, hypnotics, anxiolytics (15); opioids (7); stimulants (6); cannabis (4); cocaine (2); mixed (3)	self-referral (75); coworker or family member (20); other (6)

**Table 3:** Characteristics of monitoring.

Year, Study	Name of the Health Program	Recommended Type of Treatment	Monitoring Elements				Monitoring Outcomes	
			Start of Monitoring	Biological Monitoring	Monitoring at Work	Other Agreements	Follow-Up: % Abstinence	Follow-Up: % Work Retention
1982, Herrington et al. [20]	Impaired Physician Treatment Program	inpatient (95%)	after treatment completion	urine	yes	participate in Alcoholics or Narcotics Anonymous groups, attendance of local meetings in the community, and attendance of weekly sessions of Milwaukee Doctors in Alcoholics Anonymous	0 to 3 years: 68% no relapse	0 to 3 years: 78% working
1984, Washton et al. [21]	Regent or Fair Oaks Hospital	inpatient 4–10 weeks	after treatment completion	urine		pharmacotherapy with naltrexone, group therapy, individual therapy, and family/couples therapy	0 to 1.5 years: 87% no relapse	0 to 1.5 years: 87% working
1985, Crowley [22]	Halsted Clinic	outpatient	with treatment initiation	urine	-	counseling sessions	2 years (average): 47% no relapse	-
1987, Shore [23]	rehabilitation program	-	-	urine	-	long term supervision by the medical board	-	0 to 8 years: 75% working
1991, Pelton & Ikeda [24]	California Physicians Diversion Program	-	with treatment initiation	urine	yes	attendance of two diversion group meetings per week, and two or more 12-step meetings per week	3 to 5 years: 51% no relapse 18% brief relapse	-
1992, Gallegos et al. [25]	Georgia Impaired Physicians Program continuing care program	Georgia Impaired Physicians Treatment Program	after treatment completion	urine	-	primary care physician attends to their medical needs, recovery mentor, five Alcoholics or Narcotics Anonymous meetings per week, and one Caduceus Club meeting per week	more than 5 years: 77% no relapse	more than 5 years: 91% working
1994, Roy [26]	reentry monitoring	-	after treatment completion	urine	-	group therapy	2 years (average): 81% no relapse8% brief relapse	2 years (average): 95% working
1996, Nelson et al. (1) [27]	Diversion Program	inpatient and/or outpatient	after treatment completion	urine	-	group therapy	1.5 years (average): 86% no relapse	-
1996, Nelson et al. (2) [27]	Probationary Program	inpatient and/or outpatient	after treatment completion	urine	-	group therapy	2.3 years (average): 78% no relapse	-
1997, Roth et al. [28]	special treatment program	inpatient and/or outpatient	with treatment initiation	urine	-	pharmacotherapy with naltrexone for 6 months	1.8 years (average): 60% no relapse	1.8 years (average): 60% working
1999, Paris & Canavan (1) [29]	Physician Health Program	-	after treatment completion	urine	-	participate in an aftercare group for 1 year, monthly face-to-face appointment with PHP employee, and attendance of Alcoholics Anonymous meetings	7.8 years (average): 59% no relapse	-
1999, Paris & Canavan (2) [29]	Physician Health Program	-	after treatment completion	urine	-	participate in an aftercare group for 1 year, monthly face-to-face appointment with PHP employee, and attendance of Alcoholics Anonymous meetings	7.2 years (average): 56% no relapse	-
2004, Warhaft [30]	Case Management, Aftercare and Monitoring Program	-	after treatment completion	urine and/or breath	yes	attendance at Caduceus group andattendance at mutual help group (Alcoholics or Narcotics Anonymous)	0 to 3 years: 79% no relapse	0 to 3 years: 78% working
2005, Domino et al. (1) [31]	Washington Physician Health Program	-	after treatment completion	urine	yes	frequent contact for behavioral assessment and regulatory board reports	0 to 5 years: 78% no relapse	-
2005, Domino et al. (2) [31]	Washington Physician Health Program	-	after treatment completion	urine	yes	frequent contact for behavioral assessment and regulatory board reports	more than 5 years: 68% no relapse	more than 5 years: 88% working
2005, Ganley et al. (1) [32]	North Carolina Physician Health Program	inpatient and/or outpatient	-	urine hair		meetings with volunteer monitor, participate in Alcoholics Anonymous and other self-help groups, and participate in Caduceus meetings	1 to 6 years: 65% no relapse 26% brief relapse	-
2005, Ganley et al. (2) [32]	North Carolina Physician Health Program	inpatient and/or outpatient	-	urine hair		meetings with volunteer monitor, participate in Alcoholics Anonymous and other self-help groups, and participate in Caduceus meetings	1 to 6 years: 50% no relapse 9% brief relapse	-
2006, Clark et al. [33]	Program for Recovering Nurses	mainly outpatient (69%)	with treatment initiation	urine	yes	aftercare counseling and attendance at recovery nursing support groups	3.8 years (average): 48% no relapse	3.8 years (average): 43% working
2007, Galanter et al. [34]	Committee for Physician Health	inpatient and/or outpatient	with treatment initiation	urine	yes	12-step/therapy monitor	3.4 years (average): 63% no relapse	-
2007, Knight et al. [13]	Physician Health Services	individual psychotherapy	with treatment initiation	urine	yes	attendance at Caduceus meetings or Alcoholics Anonymous	0 to 3 years: 56% no relapse	-
2008, Brewster et al. [35]	Ontario Physician Health Program	usually inpatient abstinence based 4–6 weeks	after treatment completion	urine	yes	visits to addiction medicine doctor, visits to a family doctor for routine health needs, and attendance at mutual support groups in community	5 years (exact): 71% no relapse and 14% brief relapse	-
2009, Du Pont et al. [9]	16 American Physician Health Programs	inpatient and/or outpatient	after treatment completion	urine	-	participate in Alcoholics or Narcotics Anonymous groups, participate in aftercare groups, and follow-up from Physician Health Program monitor	4.5 years (average): 78% no relapse	4.5 years (average): 72% working
2009, Fogger & McGuinness (1) [36]	Voluntary Discipline Alternative Program	inpatient and/or outpatient	after treatment completion	urine	-	-	3 years (average): 94% no relapse	2.5 years (average): 90% working
2009, Fogger & McGuinness (2) [36]	Probationary Program	inpatient and/or outpatient	after treatment completion	urine	-	attendance of 12-steps meetings at least three times per week and attendance aftercare meeting at least one time per week for one year		4.4 years (average): 96% working
2011, Merlo et al. [37]	Professional Resource Network	-	after treatment completion	urine	-	pharmacotherapy with naltrexone for at least 2 years	3.4 years (average): 91% no relapse	3.4 years (average): 82% working
2013, Angres et al. [38]	After-Care program	abstinence based 6–8 weeks	after treatment completion	urine	-	participate in Caduceus aftercare group weekly	2 years (exact): 73% no relapse	-
2013, Cross et al. [39]	Chicago treatment program for professionals	inpatient abstinence based 8–10 weeks	after treatment completion	urine	-	participate in Alcoholics or Narcotics Anonymous groups, participate in Caduceus aftercare group, and follow-up from Physician Health Program monitor	2 years (exact): 87% no relapse	-
2017, Earley et al. [40]	Injectable extended-release naltrexone	intensive outpatient	with treatment initiation	urine	-	attendance at mutual support meetings	2 years (exact): 89% no relapse	2 years (exact): 63% working
2020, Bruguera et al. [41]	Galatea Addiction Programme	inpatient (62%) and/or outpatient 7–8 weeks	with treatment initiation	urine hair	yes	participate in psychotherapy group weekly	2 years (average): 30% no relapse	-

## Data Availability

Data is contained within the supplementary material (Dataset S1: Search strategy, Dataset S2: Forest plots abstinence, Dataset S3: Forest plots work retention).

## Supplementary Materials

The following are available online at https://www.mdpi.com/2077-0383/10/2/264/s1, Table S1: PRISMA Checklist.

## Appendix A

**Table A1:** The Health States Quality Index.

Year, Study	Variables						Total (Max = 10)	Quality Index
	**1**	**2**	**3**	**4**	**5**	**6**		
1982, Herrington et al.	1	0	1	0	0	0	2	0.2
1984, Washton et al.	1	0	1	0	0	0	2	0.2
1985, Crowley	0	0	1	1	1	1	4	0.4
1987, Shore	0	1	2	1	1	0	5	0.5
1991, Pelton & Ikeda	0	0	2	1	1	0	4	0.4
1992, Gallegos et al.	1	0	1	1	1	0	4	0.4
1994, Roy	0	0	1	0	1	1	3	0.3
1996, Nelson et al. (1)	1	0	2	1	1	1	6	0.6
1996, Nelson et al. (2)	1	0	2	1	1	1	6	0.6
1997, Roth et al.	0	0	1	1	0	1	3	0.3
1999, Paris & Canavan (1)	0	0	2	1	1	1	5	0.5
1999, Paris & Canavan (2)	0	0	2	1	1	1	5	0.5
2004, Warhaft	1	0	2	0	1	0	4	0.4
2005, Domino et al. (1)	1	0	2	1	1	0	5	0.5
2005, Domino et al. (2)	1	0	2	1	1	0	5	0.5
2005, Ganley et al. (1)	1	1	2	1	1	0	6	0.6
2005, Ganley et al. (2)	1	1	2	1	1	0	6	0.6
2006, Clark et al.	1	0	2	1	1	1	6	0.6
2007, Galanter et al.	1	0	1	1	1	1	5	0.5
2007, Knight et al.	1	0	2	1	1	0	5	0.5
2008, Brewster et al.	1	1	1	1	1	2	7	0.7
2009, DuPont et al.	1	0	2	1	2	1	7	0.7
2009, Fogger & Mc-Guinness (1)	0	0	2	2	1	1	6	0.6
2009, Fogger & Mc-Guinness (2)	0	0	2	2	1	1	6	0.6
2011, Merlo et al.	0	0	2	1	1	1	5	0.5
2013, Cross et al.	1	1	2	1	1	2	8	0.8
2013, Angres et al.	0	1	1	1	0	2	5	0.5
2017, Earley et al.	1	1	1	2	2	2	9	0.9
2020, Bruguera et al.	1	1	2	1	2	1	8	0.8
**Variables**		**0**		**1**			**2**	
1. Clear definition of target population and observation period		No		Yes				
2. Use of diagnostic criteria		Symptom based Not specified		Use of diagnostic system reported				
3. Method of case selection		Not specified		Convenience sampling			Attempts all cases	
4. Type of outcome assessment		Not specified		Register Case record			Administered interview	
5. Size of study area		Not specified		Small (single site)			Broad (national or multisite)	
6. Type of prevalence measure		Range of follow-up duration		Average follow-up duration			Exact follow-up duration	
